# The Calpain, Caspase 12, Caspase 3 Cascade Leading to Apoptosis Is Altered in F508del-CFTR Expressing Cells

**DOI:** 10.1371/journal.pone.0008436

**Published:** 2009-12-24

**Authors:** Mathieu Kerbiriou, Ling Teng, Nathalie Benz, Pascal Trouvé, Claude Férec

**Affiliations:** 1 Inserm, Brest, France; 2 Faculté de Médecine et des Sciences de la Santé, Université de Brest, Brest, France; 3 Etablissement Français du Sang - Bretagne, Brest, France; 4 C.H.U. Brest, Hôpital Morvan, Laboratoire de Génétique Moléculaire, Brest, France; LMU University of Munich, Germany

## Abstract

In cystic fibrosis (CF), the most frequent mutant variant of the cystic fibrosis transmembrane conductance regulator (CFTR), F508del-CFTR protein, is misfolded and retained in the endoplasmic reticulum (ER). We previously showed that the unfolded protein response (UPR) may be triggered in CF. Since prolonged UPR activation leads to apoptosis via the calcium-calpain-caspase-12-caspase-3 cascade and because apoptosis is altered in CF, our aim was to compare the ER stress-induced apoptosis pathway between wild type (Wt) and F508del-CFTR expressing cells. Here we show that the calcium-calpain-caspase-12-caspase-3 cascade is altered in F508del-CFTR expressing cells. We propose that this alteration is involved in the altered apoptosis triggering observed in CF.

## Introduction

Cystic fibrosis (CF) is the most common lethal autosomal recessive disease in the Caucasian population. It is due to mutations in the cystic fibrosis transmembrane conductance regulator (CFTR) gene [1]–[3]. The most common mutation in CF is a missing phenylalanine at position 508 (F508del-CFTR) in the first nucleotide-binding domain of the CFTR protein. The misfolded F508del-CFTR protein does not traffic correctly to the plasma membrane and is degraded by proteasome [4]–[11]. Nevertheless, some F508del-CFTR is retained in the endoplasmic reticulum (ER) [12]. Beside the accumulation of F508del-CFTR in the ER, inflammation and infection are the major features of CF [13]. Eukaryotic cells respond to the accumulation of misfolded proteins in the ER, to inflammation and infection by activating the unfolded protein response (UPR) [14]–[15]. Some lines of evidence suggest that UPR is triggered in F508del-CFTR expressing cells due to the mutated protein itself or by exogenous factors [16]–[19]. UPR induces the transcription of genes encoding ER chaperones, protein-folding enzymes and components of the ER-associated degradation system, limiting new protein synthesis [20]–[24]. Whereas it is an adaptive process aimed to restore the ER homeostasis, it may lead to apoptosis due to an increased intracellular calcium ([Ca^2+^]_i_) content followed by the activation of the calpain (Cal-1 and -2), caspase (Csp) -12 and Csp-3 cascade [25]–[31].

Some studies have suggested perturbations in the apoptotic process in CF cell lines. Whereas it was shown that high DNA fragmentation, and likely apoptosis, is a feature of various epithelia in CF it is now admitted that failure to undergo apoptosis could contribute to the pathogenesis of the disease [32]–[35]. Therefore, according to UPR triggering in CF, our aim was to assess whether the [Ca^2+^]_i_, Cal-1, Cal-2, Csp-12 and Csp-3 cascade activation was modified in F508del-CFTR expressing cells when compared to wt-CFTR expressing cells. The comparison was also studied after UPR induction by thapsigargin (Tg). Using western blot experiment and Csp activity measurement, our results indicate that the cascade is altered in F508del-CFTR expressing cells. Indeed, we observed a decreased basal expression of Cal-1, Csp-12 and Csp-3 in delF508 expressing cells. Under stress conditions, the observed alterations were a lower expression of Cal-1, Cal-2, active form of Csp-12 and the absence of increased accumulation of the active form of Csp-3 in F508del-CFTR expressing cells. Furthermore, the Csp-12 and Csp-3 activities were decreased as well as the cells mortality. Therefore, we show that the altered apoptosis observed in CF under stress conditions (inflammation, infection) involves an altered Cal-1, Csp-12 and mostly Csp-3 activation.

## Results

**Figure 1:**
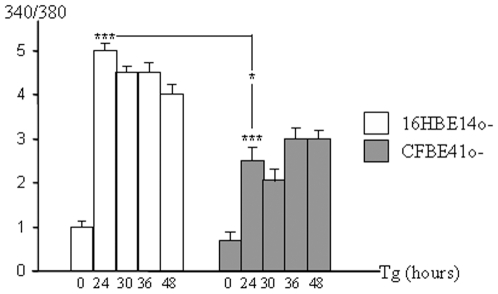
Comparison of the [Ca^2+^]_i_ between 16HBE14o- and CFBE41o- cells. 16HBE14o- and CFBE41o- cells were loaded with Fura-2 AM and fluorescence was recorded at 340 nm (saturated calcium) and 380 nm (free calcium). The 340/380 nm ratio was calculated and compared between the two cell types at increasing times of Tg treatment. Whereas no significant difference was observed between the two cell types in the absence of Tg (0 time point), [Ca^2+^]_i_ was increased in 16HBE14o- and CFBE41o- cells after 24 hours treatment. A significant lower [Ca^2+^]_i_ was observed in the F508del-CFTR expressing cells. Bars represent SEM (n = 9).

The first step of the studied cascade is a sustained increase in intracellular free [Ca^2+^] [27]. Therefore, we compared the basal free [Ca^2+^] between 16HBE14o- (wt-CFTR expressing cells) and CFBE41o- cells (F508del-CFTR expressing cells), using Fura-2 AM as a probe. No difference between 16HBE14o- and CFBE41o- cells regarding the basal [Ca^2+^]_i_ was observed (Fig. 1). 16HBE14o- and CFBE41o- cells were further submitted to Tg treatment for 24, 30, 36 and 48 hours to induce ER stress. At the 24 hours time point the [Ca^2+^]_i_ level was increased in both cell types indicating that the Tg treatment was efficient. The observed increased [Ca^2+^]_i_ remained high until 48 hours. Nevertheless, the increased [Ca^2+^]_i_ lower in CFBE41o- cells.

**Figure 2:**
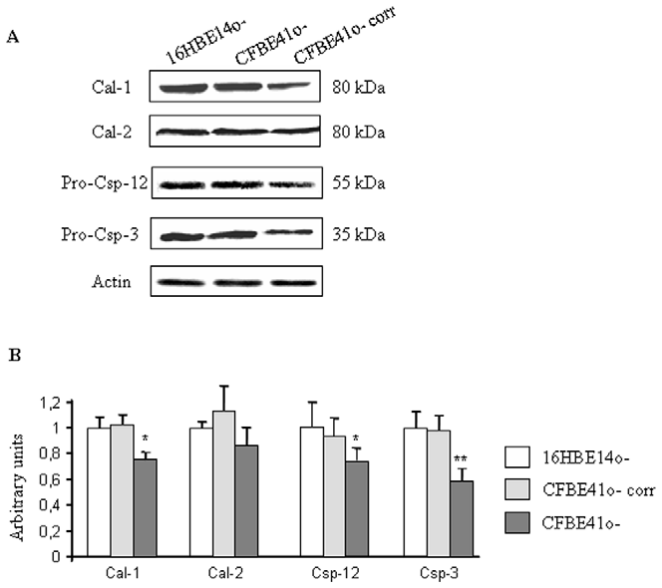
Expression of Cal-1, Cal-2, Csp-12 and Csp-3 in 16HBE14o-, CFBE41o- corr and CFBE41o- cells. **A.** Example of western blots showing the expression of Cal-1, Cal-2, pro-Csp-12, pro-Csp-3 and actin in 16HBE14o-, CFBE41o- corr and CFBE41o- cells. **B.** Histograms represent the quantification of Cal-1, Cal-2, pro-Csp-12 and pro-Csp-3 in 16HBE14o-, CFBE41o- corr and CFBE41o- cells The statistical analysis indicates a significant decreased of Cal-1, pro-Csp-12 and pro-Csp-3 in CFBE41o- cells. Bars represent SEM (n = 7).

**Figure 3:**
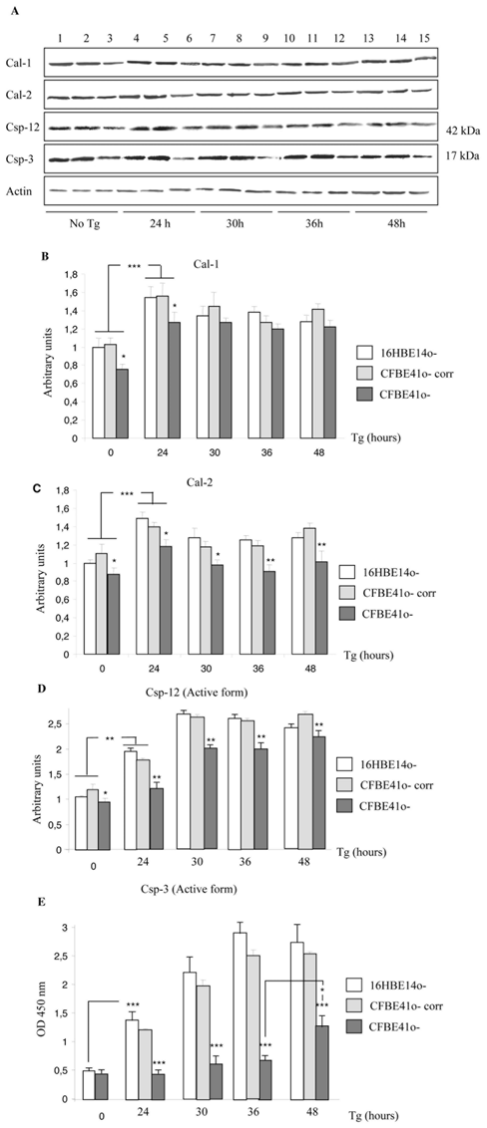
Expression of Cal-1, Cal-2, active Csp-12 and active Csp-3 in 16HBE14o-, CFBE41o- corr and CFBE41o- cells in the presence of Tg. **A.** Example of western blots showing the expression of Cal-1, active Csp-12, active Csp-3 and actin in 16HBE14o- (1, 4, 7, 10, 13), in CFBE41o- corr cells (2, 5, 8, 11, 14) and CFBE41o- cells (3, 6, 9, 12, 15) at different time point of Tg treatment. **B.** Histogram represents the quantification of Cal-1 in 16HBE14o-, CFBE41o- corr and CFBE41o- cells in the absence (0 time point) and after 24, 30, 36 and 48 hours induction by Tg. In all cell types the Cal-1 is increased in the presence of Tg. The increased expression of Cal-1 is lower in CFBE41o- cells. At the 30 time point the difference is abolished. Bars represent SEM (n = 7). **C.** Histogram represents the quantification of Cal-2. In all cell types the Cal-2 is increased in the presence of Tg. The increased expression of Cal-1 is lower in CFBE41o- cells. Bars represent SEM (n = 7). **D.** Result of the statistical analysis of the active Csp-12 expression when Tg was applied on the cells. It indicates that the active Csp-12 is overexpressed in all cell types after Tg treatment. The overexpression is lower in F508del-CFTR expressing cells. Bars represent SEM (n = 7). **E.** The-quantification of the active Csp3 was performed using an ELISA kit as described in the methods. The histogram represents statistical analysis of the optical densities (OD, 450 nm) which are proportional to the quantity of the cleaved-Csp-3 in the cell lysates. It is observed that in 16HBE14o- and CFBE41o- corr cells the cleaved form is increased after 24 hours of Tg treatment whereas it is increased in CFBE41o- cells after 48 hours. Nevertheless, the amount of the cleaved-Csp-3 remains lower in CFBE41o- cells. Bars represent SEM (n = 6).

The Cal-1, Cal-2, Csp-12 and Csp-3 expressions without Tg treatment were compared using western blottings in 16HBE14o-, CFBE41o- and corrected CFBE41o- cells (CFBE41o- corr, Fig. 2A). The statistical analysis (Fig. 2B) showed that basal Cal-1, Csp-12 and Csp-3 expressions were decreased in CFBE41o- cells, whereas the Cal-2 expression was not modified. After 24, 30, 36 and 48 hours of ER stress due to Tg, Cal-1, Cal-2, Csp-12, Csp-3 expressions were assessed in the three cell types using western blottings (Fig. 3A). Cal-1 expression was increased in all the cell types after 24 hours treatment and remained higher than in the untreated cells until 48 hours (Fig. 3B). Nevertheless, the increased Cal-1 expression was lower in the F508del-CFTR expressing cells. After 48 hours treatment the Cal-1 expression was identical in 16HBE14o- and CFBE41o- cells, indicating that F508del-CFTR expressing cells needed a longer time to respond to Tg. No difference was observed between 16HBE14o- and CFBE41o-corr cells. After 24 hours of Tg treatment, Cal-2 expression was increased in all cell types but this increased expression remained lower in CFBE41o- cells (Fig. 3C). Therefore we concluded that F508del-CFTR cells exhibit an altered Cal-2 regulation when Tg is applied on the cells. The cleavage of Csp-12 in16HBE14o-, CFBE41o- and in CFBE41o-corr cells was studied using western blottings. The statistical analysis showed that the active form of Csp-12 was increased 24 hours after Tg treatment and reached a maximum after 30 hours in all cell types (Fig. 3D). The accumulation of the active form of Csp-12 was lower in F508del-CFTR expressing cells showing that the appearance of the cleaved form of Csp-12 is delayed when F508del-CFTR is expressed. In 16HBE14o- and in CFBE41o- corr cells the accumulation of the active form of Casp-3 was increased at 24 hours of treatment and progressively increased to reach a maximum after 36 hours of treatment (Fig. 3E). This pattern of expression was correlated to a decreased full length Csp-3 expression (not shown). In CFBE41o- cells, the Csp-3 expression was lower than in 16HBE14o- and CFBE41o- corr cells. In CFBE41o- cells, no accumulation of the active form of Csp-3 was observed before 48 hours of Tg treatment, indicating an important Csp-3 alteration (Fig. 3E). The full length Csp-3 expression was not modified in these cells at any time point (not shown). Therefore, under Tg treatment, Cal-1, Cal-2 and active Csp-12 accumulation were lower and delayed in CFBE41o- cells when compared to that of 16HBE14o- and CFBE41o- corr cells. The most significant result was a delayed and lower increased expression of the active form of Csp-3 in CFBE41o- cells after Tg stress.

**Figure 4:**
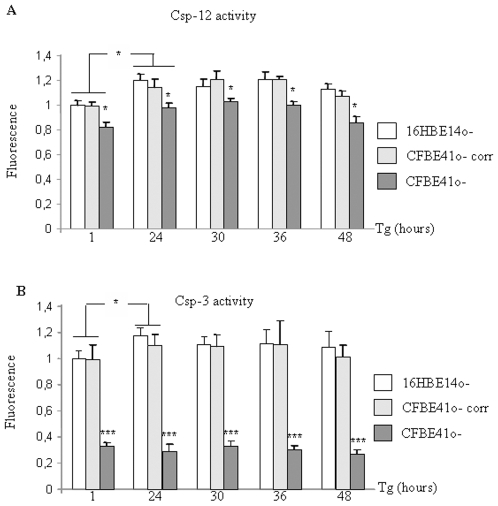
Activities of Csp-12 and Csp-3 in 16HBE14o-, CFBE41o- corr and CFBE41o- cells in the presence of Tg. Activities were measured by a fluorometric kit and the fluorescence after 1 hour incubation of 16HBE14o- cells with Tg was taken as reference. **A.** Histogram representing the results for the Csp-12 activity. The activity was increased in all cell types after 24 hours Tg. It was decreased in CFBE41o- cells at 48 hours. Bars represent SEM (n = 5). **B.** Histogram representing the results for the Csp-3 activity. It was increased in 16HBE14o- and CFBE41o- corr cells at the 24 hours time point and remained sustained until 48 hours. In CFBE41o- cells the Csp-3 activity was low and no increase among Tg incubation time was observed. Bars represent SEM (n = 6).

Csp-12 and Csp-3 activities were measured by a fluorometric kit assay. For statistical analysis, the basal activities after 1 hour of Tg treatment (time point O) were chosen as 1 unit activity. Casp-12 basal activity was found to be decreased in CFBE41o- cells when compared to that of 16HBE14o- and CFBE41o- corr cells (Fig. 4A). Nevertheless, under Tg treatment the Csp-12 activity increased in all cell types. 36 hours after Tg treatment this activity was lower in F508del-CFTR expressing cells which exhibited a level corresponding to the non treated CFBE41o- and CFBE41o- corr cells. Therefore, this result suggested that F508del-CFTR expressing cells exhibit a decreased duration of Csp-12 activation when compared to CFTR expressing cells. Csp-3 activation was observed in 16HBE14o- and in CFBE41o- corr cells 24 hours after Tg treatment. The plateau phase observed after 24 hours was likely due to a sufficient amount of active Csp-3 to clive the whole substrate, showing that the maximal activity was reached. In CFBE41o- cells the basal Csp-3 activity was lower than in 16HBE14o- and CFBE41o- corr cells (Fig. 4B). Under Tg treatment despite increased [Ca^2+^]_i_, Cal-1, Cal-2 and Csp-12 levels and despite an increased Csp-12 activity, the Csp-3 activity was hardly detected and remained very low. This result was in accordance with accumulation of the active form of Csp-3 detected by ELISA.

**Figure 5:**
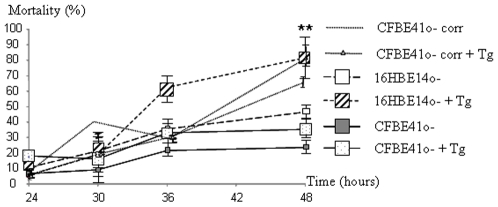
Percentage of mortality in 16HBE14o- and CFBE41o- cells in the presence of Tg. The curves represent the mortality time course under Tg treatment. In both cell types it was increased with time. Nevertheless, the mortality was higher in 16HBE14o- and CFBE41o- corr cells after 36 hours of Tg treatment (p<0.001). Bars represent SEM (n = 5).

Cal and Csp-12 activation are hallmarks of apoptosis [36]–[40]. We assessed the cell viability of 16HBE14o-, in CFBE41o- corr and CFBE41o- cells under Tg conditions. As shown in Fig. 5, the mortality was decreased in CFBE41o- cells independently of any treatment. After 36 hours of Tg treatment, the number of dead 16HBE14o- and CFBE41o- corr cells was increased and reached 80% (+/− 10%) at the 48 hours time point. Increased mortality was found in CFBE41o- cells. Nevertheless, the percentage of dead 16HBE14o- and CFBE41o- corr cells was higher than that of CFBE41o- cells (30% +/− 55%) at the 48 hours induction point. Therefore, a decreased cell death was observed in the F508del-CFTR expressing cells.

## Discussion

Mutations in the gene encoding CFTR are responsible for CF. The most common mutation F508del-CFTR, whose pathology is primarily due to a decrease in Cl permeability through the CFTR. The associated pathology is maintained by repeated lung infections, which provokes inflammatory responses that lead to lung fibrosis and respiratory failure in which lung necrosis is involved [41]. Furthermore, infected epithelial cells expressing mutant CFTR are less sensitive to apoptosis than cells expressing normal CFTR [35]. Because the released debris by cells undergoing necrosis initiate inflammatory response which is harmful in CF, it is important to understand why cells expressing mutant CFTR are more resistant to apoptosis than normal cells in the absence of infection. Consistent with this view, are the typical large DNA fragments of necrotic cells which are released by CF epithelia, increasing the viscosity of the mucus.

**Figure 6:**
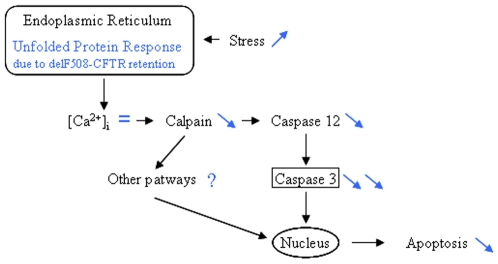
Schematic presentation of molecular mechanisms involving calpains and caspases leading to apoptosis in F508del-CFTR expressing cells. This schematic representation is adaptated from [27]. Black arrows indicate the pathway in CFTR expressing cells. In dark grey are our proposed modifications in F508del-CFTR expressing cells.

Beside the decreased Cl^−^ permeability, the misfolded F508del-CFTR is partially retained in the ER where it activates UPR which can be enhanced by infection and inflammation [12], [14]–[19]. Indeed, UPR triggering due to the mutated CFTR expression was observed in A549 cells as well as in CFBE41o- cells which were used in the present study [17], [18]. It has been proposed that multiple UPR pathways contribute to ER stress-induced cell apoptosis, although the mechanisms still remain largely unknown. Nevertheless, the involvement of the [Ca^2+^]_i_, Cal-1 and -2, Csp -12 and Csp-3 cascade is suggested [25]–[31]. Therefore, our aim was to study this cascade in wt- and F508del-CFTR expressing cells and we showed that this pathway is altered when the mutated CFTR is expressed. Indeed, we observed decreased Cal-1 and Csp expressions in CF cells. Furthermore, we showed that under stress condition which was performed to mimic the exogenous stress observed in CF, Csp decreased expressions in CF cells were accompanied by a decreased activity, mostly regarding Csp-3 (Fig. 6). Interestingly, calpains deficiency was correlated with the resistance to ER stress-induced apoptosis which was directly related to a calpain requirement for the activation of Csp-12 and our results are consistent with previous studies showing that deficient cells for the pro-Csp-12 are resistant to ER stress-induced apoptosis [27], [28]. Nevertheless the present data regarding Csp-12 have to be moderated because its involvement in UPR in human is still controversial [42]–[44]. Indeed, previous data in humans and rodents indicate that the presence of Csp-12 attenuates responsiveness to bacterial invasion and confers a survival disadvantage [45].

In conclusion, we suggest that the [Ca^2+^]_i_, Cal-1 and -2, Csp -12 and Csp-3 cascade in involved in the CF physiopathology because this cascade is altered in CF cells and that the most important alteration concerns Csp-3. Therefore, the present results provide new fields of investigation regarding the altered apoptosis observed in CF.

## Materials and Methods

### Antibodies and Drugs

Monoclonal anti-Cal-1 (9A4H8D3, ab3589) and polyclonal anti-Cal-2 (2539) were purchased from abcam and Cell Signaling, respectively. Polyclonal anti-Csp-12 (2202) and monoclonal anti-Csp-3 (9662) were purchased from Cell Signaling. Secondary antibodies and the ECL+ detection kit were purchased from Amersham. Tg (T9033) and protease inhibitor cocktail (P8849) were from Sigma. Csp-12 Fluorometric Assay Kit (K139-100) and Csp-3 Fluorometric Assay Kit (K105-100) were from Biovision and were used to study the activity of these Csp.

### Cell Culture and Treatment

Pr. D.C. Grüenert (San Francisco, CA, U.S.A) kindly provided the normal human bronchial polarized epithelial cells 16HBE14o- expressing the normal CFTR, their CF counterparts CFBE41o- expressing the F508del-CFTR and the corrected CFBE41o- cells [46]–[50]. The cells were cultured in Eagle's minimal essential medium (MEM) supplemented with 10% FBS and glutamine on an extracellular matrix (CellBind Corning, Bioblock). To induce ER calcium depletion and the activation of the [Ca^2+^]_i_, Cal-1, Cal-2, Csp-12 and Csp-3 cascade, cells were treated with 5 µM Tg for 24, 30, 36 and 48 hours.

### Viability Assays

Cell viability after Tg treatment was determined by trypan blue exclusion. Cells were trypsinized, resuspended in phosphate buffer saline (PBS), incubated with 0.4% trypan blue (prepared in PBS) for 8 min and counted (at least 600) for calculation of percentage of residual cell viability, as previously described [26].

### Fura 2-AM Measurement

[Ca^2+^]_i_ in 16HBE14o- and CFBE41o- cells with and without Tg treatment was measured using the Ca^2+^-sensitive fluorescent probe fura-2 acetoxy-methyl ester (Fura-2 AM, Invitrogen). Cells were loaded for 45 min in the dark with 2.5 µM Fura-2 AM at 37°C. They were rinsed twice and incubated in Tyrode solution for 20 min at 37°C. Fluorescence was recorded using the fluorescence microscope (IX-71, Olympus, France) at 340 nm (saturated Ca^2+^) and 380 nm (free Ca^2+^) and was further analyzed using Fluostar software (IMSTAR, France). For each experiment, 20–30 cells were observed, the responses were compared to the controls obtained with untreated cells and the [Ca^2+^]_i_ concentration was calculated as previously described [51].

### Protein Extraction and Western Blotting

Cell lysates were prepared from 16HBE14o- and CFBE41o- cells in lysis buffer (50 mM Tris HCl, 100 mM NaCl, 1% Triton X-100) in the presence of protease inhibitor cocktail (5 µL/mL; 1.1 µM leupeptin, 0.7 µM aprotinin, 120 µM PMSF, 1 µM iodoacetamide, 0.7 µM pepstatin and 1 mM DIFP). Protein concentrations were determined by the method of Lowry et al. using bovine serum albumine as a standard [50]. Equal amounts of total proteins for each sample were submitted to 37°c heating and subjected to SDS/PAGE (12%). After blotting, the membranes were saturated in PBS containing 0.2% fat free milk, incubated overnight (4°c) with anti-Cal-1 (1/1000), anti-Cal-2 (1/1000), anti-Csp-12 (1/500) and anti-Csp-3 (1/500) antibodies and with appropriate secondary antibodies (1/5000). Revelations were performed using an ECL+ detection kit. Densitometric analysis of the signals was performed using a GelDoc 2000 apparatus (Biorad) and each value was normalized by the total amount of loaded proteins per lane which was estimated after coomassie blue staining of the membrane and by the actin signal obtained on the same membranes, which did not vary among cell types.

### Cleaved Csp-3 Detection

A solid phase sandwich enzyme-linked immunosorbent assay (ELISA, Cell Signaling) was used to detect and quantify the cleaved Csp-3, according to the manufacturer's instructions. In this assay, a total Csp-3 antibody is coated onto microwells. After incubation with the cell lysates, cleaved and uncleaved Csp-3 is captured. Following extensive washing a biotinylated cleaved-Csp-3 antibody is used to specifically detect this form of the protein. The magnitude of optical density (OD, 450 nm) of the coloured reaction is proportional to the quantity of cleaved-Csp-3 (17 kDa). All time points were performed in triplicate and the experiment was performed three times.

### Caspases Activities Assays

Csp-12 and Csp-3 activities were measured by a fluorometric kit assay according to manufacturer's instructions. In brief, cells were lysed and the proteins (20 µg) were incubated with the Csp-12 substrate ATAD-AFC (50 µg) or with the Csp-3 substrate DEVAD-AFC (50 µg), for 2 hours at 37°C. Samples are transferred in black bottom 96 wells microplates and were read in a fluorescence microplate reader (Fluoroskan Ascent; Labsystems). The non-clived substrate emission was 400 nm (blue) and the clived substrate emission was 505 nm (green). The control reactions were performed with no protein in the wells and by omitting the substrate. Experiments were performed in triplicate.

### Statistical Analysis

Data are shown as mean ± standard error of mean (SEM). Differences between experimental groups were evaluated by a two-tailed unpaired Student's *t* test. p<0.05 was considered significant (*), p<0.001 was considered very significant (**) and p<0.0001 was considered highly significant.
